# 3D-Printed Capillary Circuits for Calibration-Free Viscosity Measurement of Newtonian and Non-Newtonian Fluids

**DOI:** 10.3390/mi9070314

**Published:** 2018-06-21

**Authors:** Sein Oh, Sungyoung Choi

**Affiliations:** Department of Biomedical Engineering, Kyung Hee University, 1732 Deogyeong-daero, Giheung-gu, Yongin-si, Gyeonggi-do 17104, Korea; hottaek_90@khu.ac.kr

**Keywords:** viscometer, 3D printing, capillary circuit, microfluidics, Newtonian fluid, non-Newtonian fluid

## Abstract

Measuring viscosity is important for the quality assurance of liquid products, as well as for monitoring the viscosity of clinical fluids as a potential hemodynamic biomarker. However, conventional viscometers and their microfluidic counterparts typically rely on bulky and expensive equipment, and lack the ability for rapid and field-deployable viscosity analysis. To address these challenges, we describe 3D-printed capillary circuits (3D-CCs) for equipment- and calibration-free viscosity measurement of Newtonian and non-Newtonian fluids. A syringe, modified with an air chamber serving as a pressure buffer, generates and maintains a set pressure to drive the pressure-driven flows of test fluids through the 3D-CCs. The graduated fluidic chambers of the 3D-CCs serve as a flow meter, enabling simple measurement of the flow rates of the test fluids flowing through the 3D-CCs, which is readable with the naked eye. The viscosities of the test fluids can be simply calculated from the measured flow rates under a set pressure condition without the need for peripheral equipment and calibration. We demonstrate the multiplexing capability of the 3D-CC platform by simultaneously measuring different Newtonian-fluid samples. Further, we demonstrate that the shear-rate dependence of the viscosity of a non-Newtonian fluid can be analyzed simultaneously under various shear-rate conditions with the 3D-CC platform.

## 1. Introduction

Measuring viscosity is important for assessing the quality of liquid products [1,2,3,4,5], optimizing the performance of microfluidic devices [6,7], and monitoring the viscosity of clinical fluids as a hemodynamic biomarker [8]. Testing the viscosity of liquid products is an indispensable process to maximize production efficiency and to screen formulation candidates across diverse industrial fields [1,2,3,4,5]. Whole blood viscosity has a significant correlation with Alzheimer’s disease and can be used as a potential biomarker for the monitoring and control of the disease [8]. Hyperviscosity syndrome is a combination of clinical symptoms (e.g., mucosal hemorrhage, visual abnormalities, or neurological disorders) caused by increased blood viscosity. It can be diagnosed by measuring whole blood viscosity or plasma viscosity, and treated by plasmapheresis and blood transfusion [9].

Two types of viscometers are commonly used for such applications and categorized into cone-plate viscometers [10] and capillary viscometers [11]. Cone-plate viscometers utilize a rotating cone that applies a shearing force and measures the resistance of a test fluid between the cone and a stationary plate [10]. Although cone-plate viscometers allow viscosity measurement over a wide range of viscosities and shear rates, their use at the sampling point for rapid and on-site viscosity measurement is typically limited by low measurement throughput and the requirement of bulky and costly equipment. Capillary viscometers are based on the Hagen-Poiseuille law and determine the viscosity of a test fluid from a measured flow rate under a given pressure condition or a measured pressure under a given flow-rate condition [11]. While relatively small and simple to operate, capillary viscometers require expensive, high-precision sensors and actuators for accurate viscosity measurement, thereby, increasing the cost of analysis.

Recently, miniaturized viscometers have been developed by adopting the advantages of microfluidic technologies, including low sample consumption, cost-effectiveness, small device footprint, and ease-of-use [12], and can be categorized as being based on a single microchannel [13,14], a falling ball [15], droplet microfluidics [16,17], a microfluidic comparator [18,19,20], or an optical tweezer [21]. The microchannel approach utilizes the relationship between the flow rate and viscosity of a test fluid flowing through a microchannel under a given capillary pressure [13,14]. Falling-ball viscometers measure the settling velocity of a ball falling in a test fluid that is correlated to the viscosity of the fluid of interest [15]. In droplet-based viscometers, micro-droplets are generated through a flow-focusing device and the length of the droplets, which is a function of rheological parameters, determines the viscosity of an aqueous or non-aqueous fluid [16,17]. The comparator approach uses a parallel microchannel in which the co-flowing laminar streams of two fluids, namely, a reference fluid with a known viscosity and a test fluid with an unknown viscosity, can form different interfacial positions or equilibrium states depending on their viscosities [18,19,20]. Thereby, the stream-width ratio or the flow-rate ratio of the two fluids determines the relative viscosity of the test fluid. An optical tweezer has been used to measure viscosity by monitoring the trajectory of an optically-trapped bead [21]. Although this method enables accurate viscosity measurement with low sample consumption in a few microliters per measurement, it typically requires complex and costly optical setup and lacks the ability to perform multiplexed viscosity measurement. The above approaches have been proven effective to analyze various kinds of fluids, such as whole blood [15,18,19], blood plasma [13], and polymer products [14], but there are limitations that impede the wide-spread use of these approaches in industrial and clinical applications. The microchannel approach requires cumbersome microscopic flow observation for viscosity measurement. The falling ball approach lacks sufficient controllability to generate a range of shear rates for the analysis of a non-Newtonian fluid. The comparator and droplet-based viscometers rely on bulky and high-precision pumps for the stable and accurate generation of co-flowing laminar streams and micro-droplets, respectively, which increases the size and cost of the overall system. To address these challenges, our group has recently developed 3D-printed capillary circuits (3D-CCs) that consist of parallel capillary channels for simple viscosity measurement [22,23]. The 3D-CCs, though enabling equipment-free and multiplexed viscosity measurement, is highly dependent on the accuracy of a reference-fluid viscosity, requiring its additional calibration for accurate viscosity measurement.

**Figure 1:**
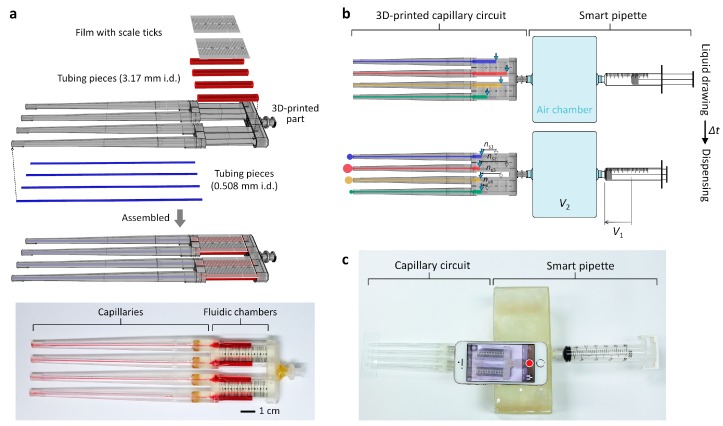
Working principle of the 3D-printed capillary circuit (3D-CC) platform for equipment- and calibration-free viscosity measurement. (**a**) (Top) Fabrication process of the 3D-CC having four identical fluidic channels for the multiplexed analysis of Newtonian fluids. (Bottom) Optical image of the fabricated 3D-CC filled with red ink for visualization; (**b**) schematic of the operation process: (top) Scale reading after withdrawing test liquids and (bottom) expelling the liquids by fully depressing the plunger. The volumetric flow rate (*Q*) of each test fluid can be measured by the number of scale ticks (*n*_s_) counted for a given time. Knowing the *Q*, capillary dimensions, and drop in pressure through each capillary, the viscosities of the test liquids can be simultaneously determined without cumbersome calibration and expensive equipment; (**c**) experimental setup for multiplexed viscosity measurement using the 3D-CC platform composed of the 3D-CC and smart pipette.

Here, we report new 3D-CCs that enable equipment- and calibration-free viscosity measurement of Newtonian and non-Newtonian fluids (Figure 1). The 3D-CC platform enables simple flow-rate measurement using the graduated fluidic chambers of the 3D-CCs and portable generation of a set pressure using a modified syringe, called a ‘smart pipette’, that is powered by hand. Based on the Hagen-Poiseuille law, the viscosities of test fluids flowing through the capillaries of the 3D-CCs can be calculated from the measured flow rates under a set pressure condition. The number of test fluid samples to be simultaneously analyzed can be augmented simply by adding more fluidic channels. We demonstrated the multiplexed capability of the 3D-CC platform by simultaneously measuring four Newtonian fluid samples within two minutes and comparing the measurement results with a conventional cone-plate viscometer. We also demonstrated that the 3D-CC platform enables simple analysis of a non-Newtonian fluid under multiple shear-rate conditions, proving the potential of the 3D-CC platform for rapid and on-site viscosity analysis of Newtonian and non-Newtonian fluids.

## 2. Materials and Methods

### 2.1. Device Fabrication

The 3D-CCs were fabricated by assembling a 3D-printed housing and cut tubing pieces to be used as the capillaries and fluidic chambers of the 3D-CCs (Figure 1). The 3D-printed parts were fabricated using a stereolithography-based 3D printer (DWS Systems, Thiene, Italy). The details of fabrication using stereolithography can be found in previous literature [24,25]. Tygon^®^ tubings, with an inner diameter of 0.508 and 3.17 mm (Cole-Parmer, Vernon Hills, IL, USA), were cut into pieces to serve as the capillaries and fluidic chambers of the 3D-CCs, respectively. The air chamber, with an inner volume of 1.27 L, was fabricated using a 3D printer. Luer-Lock fittings were attached to the ends of the chamber for reversible and airtight interconnection between a 3D-CC and a syringe (Figure 1c). A film mask with scale ticks was fabricated, cut into pieces, and attached to the assembled 3D-CC for volume indication (Figure 1).

A microfluidic viscometer for comparison with the 3D-CC for analysis of a non-Newtonian fluid was fabricated by photolithography and polydimethylsiloxane (PDMS) replica molding processes as previously reported (Figure S1) [22]. The comparator is composed of an inlet device and an outlet device. The inlet device has two inlets, a junction channel, and two outlets, and the outlet device has two inlets and one outlet. The devices were connected through cut tubing pieces, having an inner diameter of 0.508 mm and a length of 20 cm. The microfluidic comparator is based on the comparison of two different fluid streams (i.e., a test fluid and a reference fluid) in the junction channel. The ratio of their viscosities can be determined by the ratio of their flow rates at the equilibrium state, where the interfacial position of the fluids is stationary in the junction channel. This approach requires additional calibration for the reference-fluid viscosity to calculate the absolute viscosity of the test fluid.

### 2.2. Sample Preparation and Analysis

A glycerol solution was purchased from Sigma-Aldrich (St. Louis, MO, USA) and diluted in distilled water at various concentrations (50 to 64.5 wt %) for the preparation of Newtonian fluids of different viscosities. The viscosities of the glycerol-water mixtures were determined using a cone-plate viscometer (Brookfield AMETEK Inc., Middleborough, MA, USA). A xanthan gum solution was obtained from Sigma-Aldrich and diluted in distilled water at a concentration of 0.1 wt % for analysis of a non-Newtonian fluid. All experiments with the 3D-CCs were recorded using a smartphone and changes in liquid level were measured during 15 s. The volumetric flow rate in each channel of the 3D-CCs was calculated by multiplying the cross-sectional area of the fluidic chamber by the number of measurement ticks counted per unit time. The microfluidic comparator was operated using two syringe pumps (KD Scientific, Holliston, MA, USA), and the flow rates of the test and reference fluids were manually adjusted to determine the equilibrium state.

### 2.3. Computational Fluid Dynamics Simulation

The pressure drop profile along the channel of the 3D-CC for multiplexed analysis of Newtonian fluids was analyzed using COMSOL Multiphysics (COMSOL, Burlington, MA, USA) to verify the design criterion for the pressure-drop ratio between the capillary and fluidic chamber. The wall shear rate and pressure drop along the length of each capillary of the 3D-CC for analysis of a non-Newtonian fluid were also analyzed using COMSOL Multiphysics to determine capillary lengths to achieve desired shear-rate conditions. Three-dimensional finite element models were created in the same dimensions as the 3D-CCs and solved using the incompressible Naiver-Stokes equation. The inlet boundary condition was set to a constant pressure, while the outlet boundary condition was set to atmospheric pressure. All other boundary conditions were set with no-slip boundary conditions.

## 3. Results and Discussion

### 3.1. Measurement Principle

The 3D-CC platform is composed of a 3D-CC and a smart pipette to measure the volumetric flow rates (*Q*) of test fluids discharging through the channels of the 3D-CC and to pump the fluids at a fixed pressure drop, respectively (Figure 1). The channels of the 3D-CC are arranged in a successive structure of a capillary and a fluidic chamber. The scales marked on the fluidic chamber enable accurate measurement of the liquid volume discharging through each channel over a given time (∆*t*), thereby, determining *Q*. The smart pipette has an air chamber serving as a pressure buffer, thereby, generating a fixed pressure condition [26,27,28,29]. The Hagen-Poiseuille law is the basis of the 3D-CC platform to measure viscosity without a reference fluid [30]:

(1) $$Q=\frac{\pi r^{4}\Delta P}{8\;\mu L}$$

where ∆*P* is the pressure drop through each capillary of the 3D-CC, *r* is the capillary radius, µ is the fluid viscosity, and L is the capillary length. Test fluids of an unknown viscosity are filled and dispensed through the channels of the 3D-CC under a fixed pressure-drop condition (Figure 1b). The *Q* of each test fluid can be measured by the number of scale ticks (*n*_s_) for ∆*t*. Knowing the *Q*, capillary dimensions and drop in pressure through each capillary, the viscosities of test fluids can be simply calculated using Equation (1).

There are two design parameters that affect viscosity measurement accuracy. The first parameter is the volume setting of the smart pipette. Depressing the syringe plunger in a set volume (*V*_1_) compresses the air inside the air chamber and generates a desired pressure (*P*). Since the product of pressure and volume is a constant at a constant temperature based on Boyle’s law, *P* can be defined as *P* = (*V*_1_/*V*_2_)*P_atm_*, where *V*_2_ is the volume of compressed air. However, a continual expansion in *V*_2_ occurs due to the discharging volume of liquid, *V*_3_, thereby, affecting *P*. Thus, *V*_1_ and *V*_2_ should be sufficiently larger than *V*_3_ to maintain *P*. The smart pipette was, thus, designed with a large air chamber volume of 1.27 L, at which *V*_3_ becomes negligible. At (*V*_1_)_min_ = 20 mL, *V*_2_ = 1.27 L and (*V*_3_)_max_ = 0.5 mL, the smart pipette can, theoretically, maintain *P* within 99.96%. A pressure sensor can be further integrated into the smart pipette for accurate pressure measurement.

The resistance ratio between the capillary and fluidic chamber of the 3D-CC is another design parameter for accurate viscosity measurement. Under a fixed *P*, the applied pressure drops along the capillary and fluidic chamber. In the serial connection, the upstream fluidic chamber resistance (*R*_f_) can affect the downstream ∆*P*, i.e., the pressure drop through the capillary. During liquid dispensation, *R*_f_ decreases due to a decrease in the length of the liquid filling in each fluidic chamber. The decreasing *R*_f_ can result in changes in ∆*P*, leading to an inaccurate viscosity measurement. To maintain the pressure drop through the capillary at *P* regardless of *R*_f_, it is necessary to have a capillary, *R*_c_ > 410*R*_f_, as previously reported [22], where *R*_c_ is the capillary resistance. Under this condition, it can be assumed that *P* drops only in the capillaries of the 3D-CC. *Q* can be measured by multiplying the cross-sectional area of the 3D-CC chamber and the length difference of the liquid meniscuses moved during liquid dispensation. As a result, Equation (1) can be rewritten as:

(2) $$\mu =\frac{\pi r^{4}}{8LQ}P=\frac{\pi r^{4}\Delta t}{8L\left(\pi r_{fl}^{2}dn_{s}\right)}\left(\frac{V_{1}}{V_{2}}\right)P_{atm}$$

where *r_fl_* is the radius of the fluidic chamber and *d* is the distance between adjacent scale ticks. Therefore, simply pushing the plunger of the smart pipette by *V*_1_ and counting *n*_s_ during Δ*t* enables accurate viscosity measurement by calculating Equation (2), with a set *P*, known geometric dimensions, and measured *n*_s_. The concept of connecting multiple capillaries and generating fluid flows through the capillaries using the portable pressure generator enables multiplexed viscosity analysis of different fluid samples, equipment-free viscosity measurement and viscosity analysis of a non-Newtonian fluid over a range of shear rates, and provides ease of use similar to a conventional pipette, as well as a new perspective for 3D-printed fluidic sensors.

### 3.2. Multiplexed Analysis of Newtonian Fluids

**Figure 2:**
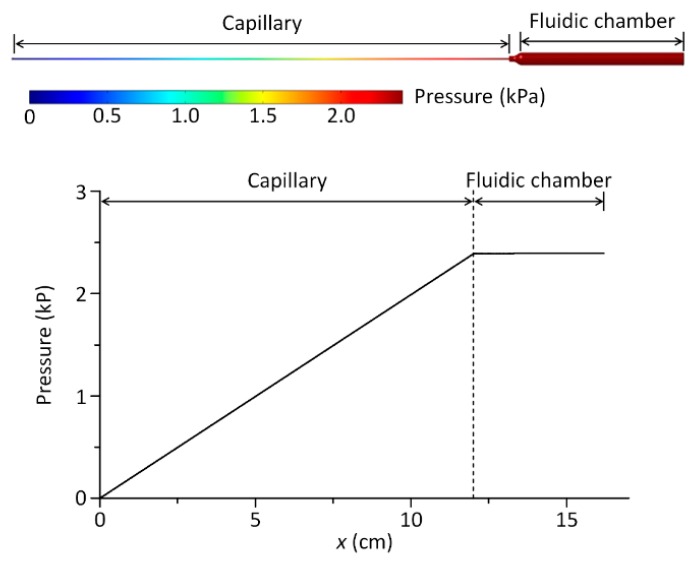
Computational fluid dynamics simulation showing the pressure profile along the fluidic channel of the 3D-CC for multiplexed analysis at *µ* = 11.0 cP and *P* = 2.4 kPa. Compared to the capillary, the drop in pressure along the fluidic chamber is negligible.

**Figure 3:**
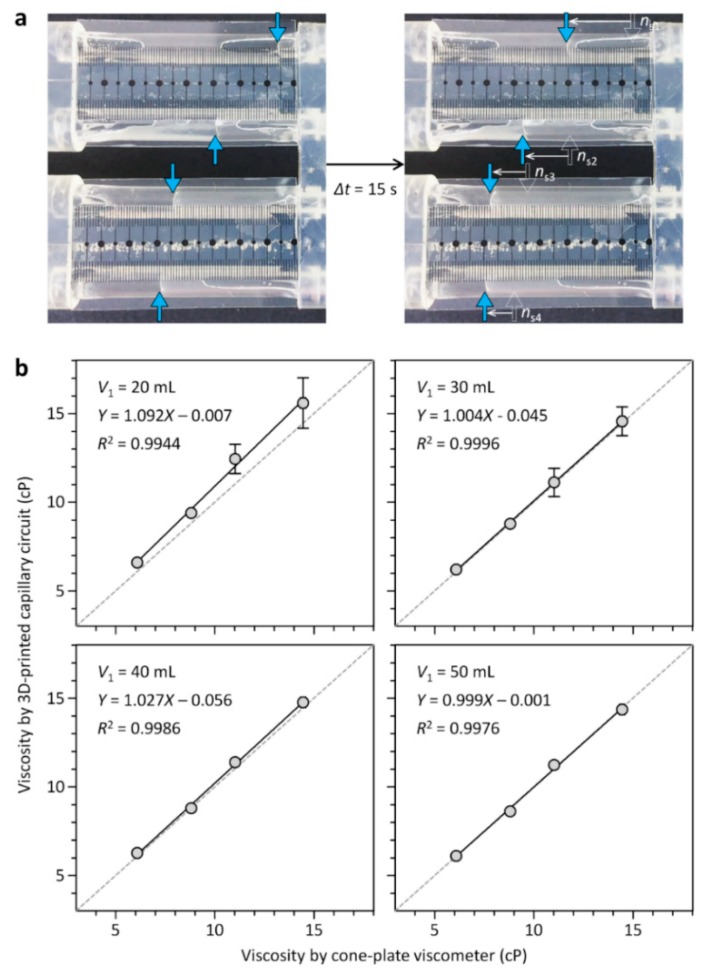
Multiplexed analysis of Newtonian fluids using the 3D-CC, having four identical fluidic channels. (**a**) Glycerol-water mixtures with different viscosities were analyzed using the 3D-CC platform at *V*_1_ = 30 mL. Their viscosities were 6.1, 8.8, 11.0, and 14.4 cP (from top to bottom) as determined by the cone-plate viscometer. The blue arrows indicate meniscuses; (**b**) effect of *V*_1_ on viscosity measurement. Solid lines denote linear regressions, while dotted lines represent a unity slope. Error bars: s.d. (*n* = 3).

**Figure 4:**
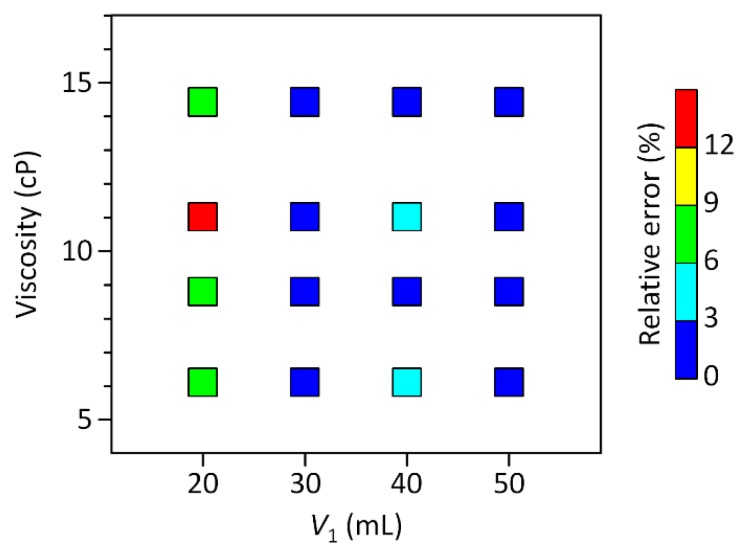
Relative errors of the 3D CC-based viscosity measurement results in Figure 3b as a function of *V*_1_ and the absolute viscosities measured using the cone-plate viscometer. Optimal operation conditions for accurate viscosity measurement can be determined from the color map for further experiments.

We first tested the ability of the 3D-CC platform for multiplexed analysis of Newtonian fluids. The 3D-CC for multiplexed analysis of Newtonian fluids was fabricated by incorporating 12 cm-long Tygon^®^ tubing pieces (inner diameter: 0.508 mm) as a capillary, 3.9 cm-long Tygon^®^ tubing pieces (inner diameter: 3.17 mm) as a fluidic chamber, and a scale-patterned film into a 3D-printed part (Figure 1). Under this condition, the ratio of *R*_c_ and *R*_f_ was greater than 4000 and the pressure drop along the fluidic chamber was negligible, satisfying the above design criteria (Figure 2). Glycerol-in-water mixtures in different weight ratios were used as a model system for Newtonian fluids of different viscosities. Their viscosities of the glycerol solutions were measured using a commercial cone-plate viscometer and compared with the results obtained from the 3D-CC. Viscosity measurements were performed by a simple two-step process: Withdrawing and dispensing test fluids through the channels of the 3D-CC. At *P* = 2.4 kPa and ∆*t* = 15 s, *n*_s_ highly depended on the viscosity of a fluid discharging through each channel of the 3D-CC (Figure 3a). As shown in Figure 3b, the viscosity data obtained using the 3D-CC in the range of *V*_1_ (30 to 50 mL), which corresponds to the range of *P* (2.4 to 4.0 kPa), had good agreement with those measured using the cone-plate viscometer. We note that the coefficient of variation (CV) for *V*_1_ = 20 mL and *µ*_c_ = 14.4 cP was 9.1%, which was significantly higher than the averaged CV of 3.5% for other *V*_1_ and *µ*_c_ conditions (range; 0–6.7%) (Figure 3b), where *µ*_c_ is the viscosity measured using the cone-plate viscometer. This is likely due to the low count of *n*_s_ at *V*_1_ = 20 mL and *µ*_c_ = 14.4 cP, which was 6.7 ± 0.6. If *n*_s_ is too low, then the measured *µ* may not be representative of the actual *µ* due to an increased effect of stochastic variables. It was, thus, necessary to use *n*_s_ ≥ 9 to ensure reliable viscosity measurement. In addition, the viscosity value (15.6 ± 1.4 cP) measured using the 3D-CC at *V*_1_ = 20 mL and *µ*_c_ = 14.4 cP was higher than *µ*_c_ (14.4 ± 0.1 cP) (Figure 3b). This might be due to the low shear rate condition (108.7 s^−1^) for viscosity measurement under which fluid-wall interactions and gravitational effects can become dominant. Such additional effects make the test fluid appear to have a high viscosity. As shown in Figure 4, *V*_1_ or the corresponding shear rate condition is an important operational parameter for accurate viscosity measurement at fixed capillary geometry and *V*_2_. The shear rate condition for 3D-CC operation should be higher than 108.7 s^−1^ for accurate viscosity measurement.

### 3.3. Analysis of a Non-Newtonian Fluid versus Shear Rate

**Figure 5:**
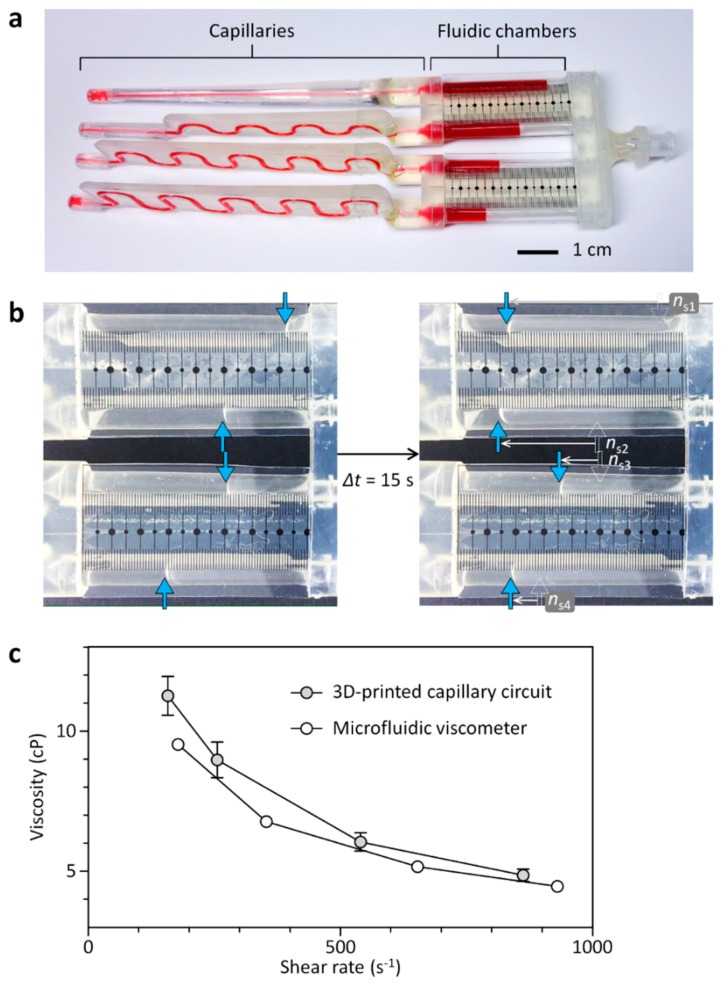
3D-CC for analysis of a non-Newtonian fluid under multiple shear-rate conditions. (**a**) Optical image of the fabricated 3D-CC having four-different fluidic channels in length. The lengths were 8.5, 10.9, 15.5, and 20.0 cm (from top to bottom). The channels of the 3D-CC are filled with red ink for visualization; (**b**) a xanthan gum solution, which served as a non-Newtonian fluid, was analyzed using the 3D-CC at different shear-rate conditions of 864, 541, 257, and 159 s^−1^ (from top to bottom); (**c**) analysis results for the xanthan gum solution using the 3D-CC and microfluidic viscometer. Error bars: s.d. (*n* = 3).

**Figure 6:**
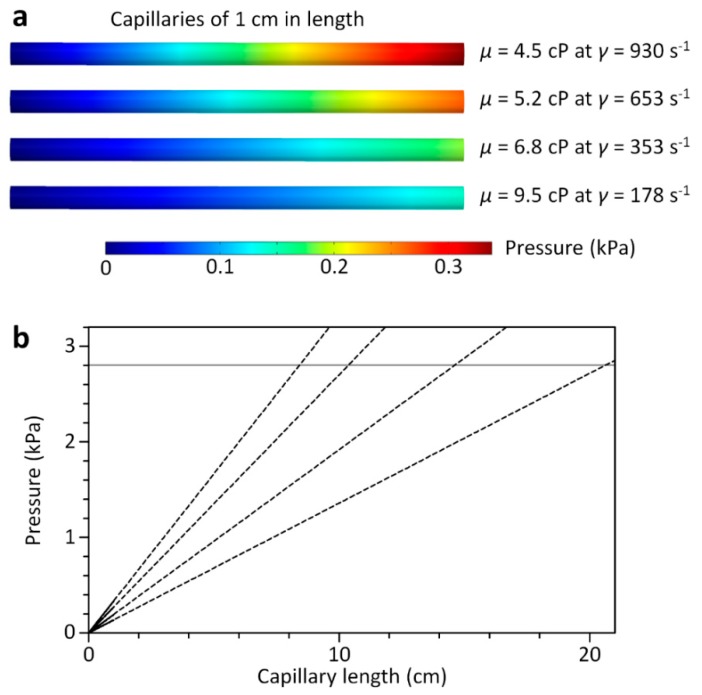
Computational fluid dynamics simulation for determining the capillary lengths of the 3D-CC for analysis of the xanthan gum solution under multiple shear-rate conditions. (**a**) Simulated pressure profiles along the capillaries (0.508 mm in inner diameter) at different *µ* and *γ* to calculate the pressure drop per unit length at each condition. The viscosities were obtained using the microfluidic comparator and varied according to the corresponding *γ*; (**b**) the different capillary lengths were determined from the relationship between ∆*P* and *L*, the Hagen-Poiseuille law, at the different *µ* and *γ* conditions. The dotted lines denote the relationships between ∆*P* and *L* for *µ* = 4.5, 5.2, 6.8, and 9.5 cP from left to right. At ∆*P* = 2.8 kPa, the capillary lengths were, respectively, determined as 8.4, 10.4, 14.6, and 20.6 cm that can, subsequently, generate the corresponding *γ* conditions for the 3D-CC.

We next tested the capability of the 3D-CC platform to analyze a non-Newtonian fluid, xanthan gum, at different shear-rate conditions. The 3D-CC for analysis of a non-Newtonian fluid was fabricated using Tygon^®^ tubing pieces (inner diameter: 0.508 mm) in different lengths for the generation of different shear-rate conditions (Figure 5a). To generate a range of shear rates (150 to 900 s^−1^), the viscosities of a 0.1 wt % xanthan gum solution were first measured at *γ* = 178, 353, 653, and 930 s^−1^ using a conventional microfluidic comparator (Figure S1); where *γ* is the wall shear rate. With the measured viscosities, we numerically calculated ∆*P* per unit capillary length (1 cm) required for the operation of the 3D-CC (Figure 6a). The capillary lengths of the 3D-CC were then determined using Equation (1) at ∆*P* = 2.8 kPa and *r* = 0.254 mm, which were 8.4, 10.4, 14.6, and 20.6 cm for *γ* = 930, 653, 353, and 178 s^−1^, respectively (Figure 6b). For convenience of fabrication, *L* was set to 8.5, 10.9, 15.5, and 20.0 cm, and *r_fl_* of the 3D-CC was set to 1.59 mm. The dimensional conditions satisfy the design criteria described above, while the resistance ratio between the capillary and fluidic chamber was greater than 4000. Depressing the plunger of the smart pipette, set at *V*_1_ = 35 mL, simultaneously generated four-different shear-rate conditions according to capillary length (Figure 5b), which corresponded to 864, 541, 257, and 159 s^−1^ for *L* = 8.5, 10.9, 15.5, and 20.0 cm, respectively. For all conditions, *n*_s_ at ∆*t* = 15 s was greater than 9, satisfying the design criteria described above. As shown in Figure 5c, the viscosity results measured using the 3D-CC were closely matched with those obtained using the microfluidic comparator, demonstrating the effectiveness of the 3D-CC platform for simultaneously testing multiple shear-rate conditions. Compared with current state-of-the-art viscometers [13,14,15,16,17,18,19,20,21,22], the proposed 3D-CC platform allows rapid, simple, and intuitive operation without bulky, complicated, high-precision equipment and additional calibration. Thus, non-experts can easily carry out the necessary procedures in a similar manner to a conventional micropipette. In addition, the scalability of the 3D-CC enables further enhancement in measurement throughput by simply adding more channels. Although xanthan gum, as a model system of a non-Newtonian fluid, was tested for proof-of-principle demonstration, the 3D-CC platform can be widely applicable for quality-control testing of liquid products and for clinical analysis of blood viscosity as a potential hemodynamic biomarker.

## 4. Conclusions

We present a new 3D-CC platform for equipment- and calibration-free analysis of Newtonian and non-Newtonian fluids. The viscometer platform allows hand-powered generation of pressure-driven flows and visual (naked-eye) determination of flow rates in parallel capillary networks. We demonstrated multiplexed viscosity measurement of Newtonian fluids of different viscosities and viscosity measurement of a non-Newtonian fluid at various shear-rate conditions. Given these novel features and the importance of viscosity measurement as a quality control means of liquid products and a diagnostic tool for evaluating hemorheological disorders, we believe that the proposed platform represents a promising approach for viscosity measurement in various industrial and clinical applications.

## Supplementary Materials

The following are available online at http://www.mdpi.com/2072-666X/9/7/314/s1, Figure S1: Schematic showing a microfluidic viscometer to measure a test fluid of unknown viscosity.
