# Supplementary material for: 3D-Printed Capillary Circuits for Calibration-Free Viscosity Measurement of Newtonian and Non-Newtonian Fluids
Source: Micromachines (Basel). 2018 Jun 21;9(7):314. doi: 10.3390/mi9070314 (PMC6082256; doi:10.3390/mi9070314)
Supplement: Supplementary file 1 [file micromachines-09-00314-s001.pdf]

Supplementary Materials: 3D-printed capillary circuits for calibration-free viscosity measurement of Newtonian and non-Newtonian fluids

Sein Oh and Sungyoung Choi*

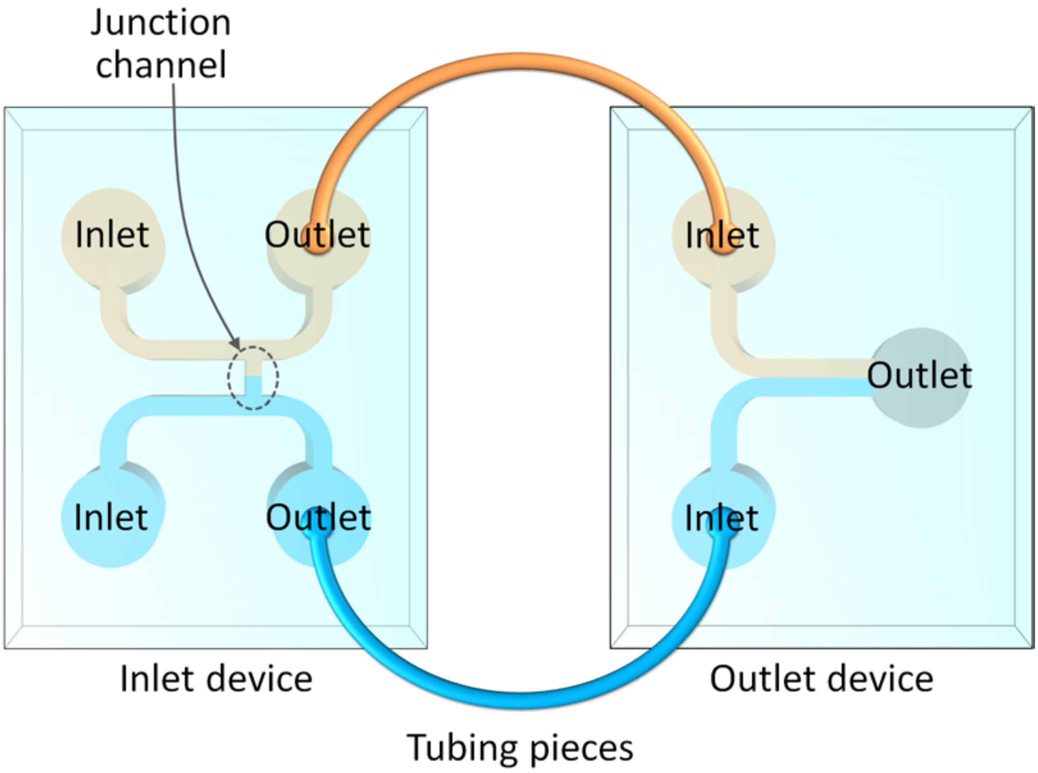

Figure S1. Schematic showing a microfluidic viscometer to measure a test fluid of unknown viscosity. The test fluid of unknown viscosity and a reference fluid of known viscosity are respectively injected into the inlet device to monitor the equilibrium state where the interfacial position of the two fluids is formed inside the junction channel. At the equilibrium state, the unknown viscosity can be determined by multiplying the flow-rate ratio of the reference and test fluids and the known viscosity of the reference fluid.
